# Keystone Veterinary Medical Association

**Published:** 1896-02

**Authors:** 


### KEYSTONE VETERINARY MEDICAL ASSOCIATION.

THE regular monthly meeting was held at the usual place, Tuesday evening, January 14, at 8 P.M. President Hart called the meeting to order, and, in the absence of the Secretary, who came in later, he appointed Leonard Pearson Secretary *pro tem.* On roll-call Drs. Bridge, Hart, Hoskins, Lintz, McAnulty, James B. Rayner, and Rhoads answered to their names. As visitors were Drs. T. B. Rayner, Chestnut Hill, Philadelphia, Pa.; Harry B. Cox, Merchantville, N. J.; J. O. George, Camden, N. J.; and James Johnston, formerly of St. Joseph, Mo., of the Bureau of Animal Industry, Philadelphia.

The Committee on Legislation reported officially the appointment of Dr. Leonard Pearson as State Veterinarian, and the President calling on the latter he referred to the preliminary meeting of the Veterinary Sanitary Board, and asked for the views of the members as to what they considered would be the best plan of dealing with the questions which were presented for consideration, specially that of tuberculosis, referring to the inadequacy of the appropriations to do any extensive work toward stamping it out.

The Association approved of a resolution which the Board had adopted in the case of suspected tuberculosis and the existence of contagious and infectious diseases, that the owner should employ a qualified veterinarian in his locality and have such animals examined, and upon the report of the veterinarian the State Board would act. This was done owing to the fact that it would be impossible for the State Veterinarian to investigate every alleged outbreak of disease throughout the State. In the discussion the influence of ventilation was freely reviewed by Drs. Bridge, James B. Rayner, Hoskins, and Pearson, bringing out many facts, showing that while ventilation was to be given important consideration, that in numerous instances the contagious and infectious character of the disease was so strongly indicated that these features must not be lost sight of. Dr. James Johnston cited a case of tuberculosis transmitted from man to a dog.

The bill for according veterinarians rank in the United States Army was read by Dr. Hoskins, and indorsed by the Association, after which a resolution prevailed that a committee of three be designated to draw up a suitable letter to be sent to the various members of the Senate and House from Pennsylvania, asking their support and consideration of the bill. The committee named were Drs. John Hart, Leonard Pearson, and W. Horace Hoskins.

Dr. W. L. Rhoads then read his paper on "The Structures of the Animal Hoof," which was listened to with a great deal of interest. The essayist brought out all the salient features connected with the growth and preservation of the foot.

Reports of cases being in order, Dr. Thomas B. Rayner reported an injury to a heifer calf occurring in September last, where a large piece of wood, about two and a half feet long, which was exhibited, had struck the animal between the eighth and ninth rib, glanced off, and, entering the abdominal cavity between the twelfth and thirteenth ribs, punctured the stomach, from which food ran out on the ground, accompanied by complete loss of power on the side of the injury. The piece of board was removed, the wound dressed with creolin and carbolic acid, and a compress-bandage adjusted, and healed kindly under this treatment. The paralysis of the hind limb remained from September until the 1st of January, when marked improvement commenced to assert itself, continuing up to the present time, the animal now being able to walk slowly upon it. Formerly the anterior face of the pastern was dragged along on the ground. Sensation remained in the limb at all times during the period of complete loss of motor power.

Mr. James B. Rayner reported a case of nearly complete serveration of the tendo-Achilles, and the animal was abandoned, turned into a meadow, receiving little or no treatment, and recovery after four months being nearly complete. Also that of a grayhound with similar injury, with recovery, after a long period of time, without special treatment.

Dr. James T. McAnulty reported a case of azoturia in a gray gelding, weighing about thirteen hundred pounds. The animal had been ill for a week undergoing treatment for toe crack, developed the disease in a stable without any preliminary exercise. A ten-drachm ball of aloes was administered, followed by three-drachm doses of bromide of potassi, and four-ounce doses of sodium sulphate to encourage the action of the physic, and a stimulating embrocation applied to the back; glycerin clysters were also used, and a second eight-drachm ball of aloes, with one-drachm of nux vomica, was administered, but purgation was not established until the fifth day. Slings were placed under the animal from which he was removed on the seventh day, when he was placed under tr. gentian, tr. nux vomica, sweet spirits ætheris nitrosis, āā three ounces; one ounce three times daily. Making a complete recovery.

Dr. Pearson reported a second case developing in a stable without any preliminary exercise, where the paralysis was confined to the shoulder; and a similar case, where the loss of power was of the shoulder, was reported by Dr. Hoskins.

The Board of Trustees reported favorably upon the application of Dr. Enoch H. Moore, and he was unanimously elected to membership.

Dr. Hoskins reported the fifth outbreak of cerebro-spinal meningitis in the same stable, occurring during the past ten years, after which, at 11 P.M., the meeting adjourned.

